# Distributions conditioned on extrapolated events via copula and extreme value theory

**DOI:** 10.1016/j.mex.2024.103017

**Published:** 2024-10-28

**Authors:** Zhankun Chen, Carl Johnsson, Carmelo D'Agostino

**Affiliations:** Department of Technology & Society, Lund University, Lund, 221 00, Sweden

**Keywords:** Copula, Extreme value theory, Traffic safety, *Copula*

## Abstract

In an interaction between road users, the proximity and speed are two interdependent dimensions which can be captured by a type of multivariate distribution called Copula. Copula requires all marginal distribution functions to be known. However, finding the marginal distribution of the proximity dimension is challenging, as its histogram usually contains several peaks. We partition the outcome space in a way that extreme value theory can be used as a tool to approximate the target marginal distribution in the tail region. In traffic safety research, such approach has the following advantages:

• The approach can approximate the distribution in the region in which the density is monotone.
• Via copula and extreme value theory, it is possible to find the conditional distribution while the conditions are not present in the data set.

## Graphical abstract

**Figure:**
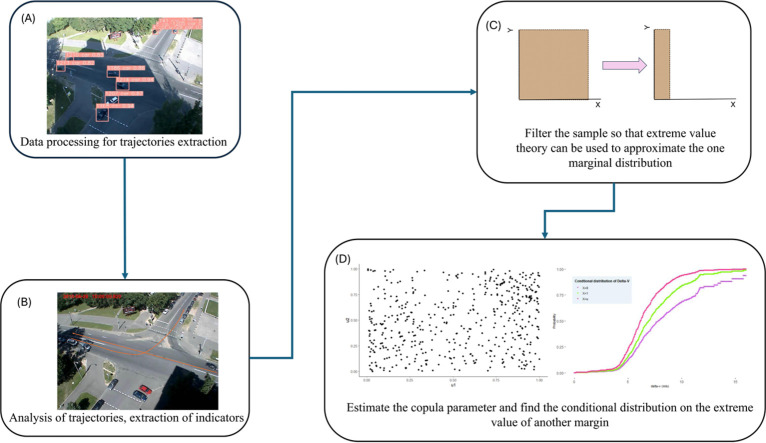

Specifications table

**Table:** 

Subject area:	Engineering
More specific subject area:	*Probabilistic model for traffic engineering*
Name of your method:	*Copula*
Name and reference of original method:	*Sklar 1959, Fonctions de répartition à n dimensions et leurs marges Annales de l'ISUP Vol. 8 Issue 3 Pages 229–231*
Resource availability:	*The data and scripts are available upon request.*

## Background

On a microscopic scale, a feasible approach to describe the road user behavior is through two interdependent dimensions: proximity and speed. For example, the distribution of speed varies when it is conditioned on different proximities. Multivariate distribution function is used to model these two dimensions simultaneously. This modeling method has applications in traffic control [1] because it enables the investigation of the dependence of road user behavior. Moreover, the method can be applied to traffic safety research, in which the road user behavior under extreme situations, such as near-accident and accidents, is of interest.

There are two technical difficulties to be addressed when modeling road users’ behaviors under extreme situations. First, it is difficult to find a marginal distribution to describe proximity. This is related to the frequent observation of multi-peaks in the histogram—suggesting that the distribution is multi-modal. Another difficulty is the absence of extreme events in the data collection period. Thus, standard method for fitting the distribution of proximity may exclude such events from the support of the distribution (formal definition of traffic events can be found in the original research article Chen et al. [2]).

Thus, we have considered using an extreme value model called peaks over threshold to filter out interactions with low proximity. It hits two birds with one stone: (1) it shrinks the support of the distribution to a region where the density is monotone; (2) it allows the extrapolation of events of unobserved proximity.

An immediate result of the method is the estimation of accident injuries in a specific site, which follows a mixed-logit model:

$$P\left(\text{Injury}\;\text{levels}|\text{accidents}\;\text{at}\;\text{site}\;X\right)=\int_{y}P\left(\text{Injury}\;\text{levels}|\text{energy}\;\text{released}\right)dF_{\text{energy}\;\text{released}\;|accident}\left(y\right)$$

In the equation above, the part first is a logistic regression model representing our knowledge. The probability of getting injury in an accident depends on many factors, among which the most influential factor is the energy released during the accident. The second part is the conditional density of the energy released during an accident, which is calibrated through the road user behavior at the site.

For all interactions at the specific site, we model the joint distribution of a two-dimensional random vector. One dimension is the spatial proximity between two vehicles. It determines how far the two vehicles are from an accident.^1^ The other dimension is the energy released during the accident. A Copula is used to capture the dependence between the two dimensions. Moreover, since accidents did not occur in collected data, $dF_{\text{energy}\;\text{released}\;|accident}\left(y\right)$ will be conditioned on extrapolated events.

The method can be extended to other fields where one variable is being influenced by the unobserved extremes of another variable. For example, investigating the entire probability distribution of grain production, instead of focusing solely on the extreme part, when it is conditioned on extreme unobserved rain falls.

## Method details

To simply describe our method: we use Generalized Pareto distribution to extrapolate events that do not exist in the data set and obtain the conditional copula distribution when it is conditioned on extrapolated events.

(1) $$F_{\text{energy}\;\text{released}\;|accident}\left(y\right)=\frac{P\left(energy\;released,accident\right)}{P\left(accident\right)}$$

Let (Ω×Ω,F⊗F,P) be the probability space in which Ω represents the space of all interactions, an interaction is then defined by the random vector $(X_{1},X_{2}):\Omega \;\times \;\Omega \mapsto \;F⊗F$, where $X_{1}$ represents the spatial proximity of the interaction and $X_{2}$ represents the (potential) released energy. There exists a unique copula that is equal in distribution to the joint distribution *F* of $\left(X_{1},X_{2}\right)$, s.t

$C\left(F_{1}\left(x_{1}\right),F_{2}\left(x_{2}\right)\right)=F\left(x_{1},x_{2}\right)|\forall \left(x_{1},x_{2}\right)$ in the continuity of F where $F_{1},F_{2}$ are the marginal distributions [3].^2^

Eq.1 can be rewritten as

(2) $$F_{X_{2}|X_{1}\leq 0}\left(y\right)=\frac{F_{X_{1},X_{2}}\left(0,y\right)}{F_{X_{1}}\left(0\right)}=\frac{C\left(F_{1}\left(0\right),F_{2}\left(y\right)\right)}{F_{1}\left(0\right)}$$

It is generally difficult to fit a suitable parametric distribution to the marginal distribution $F_{1}$. Nevertheless, it is possible to approximate the upper-tail distribution using extreme value theory [4], such that:

(3) $$\lim_{u\to x^{F}}P\left(X>x|X>u\right)\to 1-H\left(x\right)={\left(1+\gamma \frac{x-u}{\sigma }\right)}^{-1/\gamma },\;\;x^{F}=\inf\left\{x\in R:F\left(x\right)=1\right\}$$

H(x) is a generalized pareto (GP) distribution, where u is the pre-specified threshold, and σ,γ are the parameters to be estimated. The scale parameter σ>0 governs the spread of the distribution and reflects the range of events above the selected threshold. A higher scale parameter indicates greater variability. The shape parameter ξ∈R controls the tail behavior of the distribution. A non-negative shape parameter indicates that upper bound for the support of the distribution is infinity, while a negative value would indicate that the support of the distribution is bounded with an upper limit.

Since the lower tail of $X_{1}$ is of our interest, $X_{1}$ is transformed decreasing by taking the negation $\tilde{X_{1}}≔-X_{1}$ and $\tilde{X_{1}}$ is used instead of $X_{1}$ in Eq.3.

(4) $$P\left(X_{1}\leq x|X_{1}<u\right)=P\left(\tilde{X_{1}}\geq \tilde{x}|\tilde{X_{1}}>u\right)∼\bar{H}\left(T\left(x\right)\right)={\left(1+\gamma \frac{\tilde{x}+u}{\sigma }\right)}^{-1/\gamma }$$

## The probability measure for near interaction

When GP distribution is used, the outcome space is restricted to near interaction (small $X_{1}$ values). But the probability measure P(·) is associated with the event space of all interactions. It is also possible to derive the probability measure Q(·) for only near interactions, namely $P(\cdot |\Omega_{0})$ can be replaced with Q(·). First, measure Q is abstractly constructed using probability theory. The construction is done for arbitrary outcome space $\Omega_{0}\subset \Omega$ and its corresponding sigma-algebra $F_{0}\subset F$. The notations are adopted from Širjaev [5].

Consider the probability spaces (Ω,F,P) and $F_{0}\subseteq F$, where ξ is an F-measurable function. Then, $E(\xi |F_{0})$ is called the conditional expectation of ξ with respect to $F_{0}$, if

i. $E(\xi |F_{0})$ is $F_{0}$-measurble.
ii. For every $A\in F_{0}$, $\int_{A}\xi dP=\int_{A}E\left(\xi |F_{0}\right)\cdot dP$

We define the new measure $Q\left(A\right)≔\int_{A}E\left(\xi |F_{0}\right)\cdot dP$ on the measurable space $\left(\Omega_{0},F_{0}\right)$. The Radon–Nikodym derivative exists by definition and enables the change measure. Thus, on $\left(\Omega_{0},F_{0}\right)$,

$$\forall A\in F_{0},\;\;Q\left(A\right)=\int_{A}dQ=\int_{A}\frac{dQ}{dP}\cdot dP=\int_{A}E\left(\xi |F_{0}\right)\cdot dP$$

$$\;\;\Leftrightarrow \frac{dQ}{dP}=E\left(\xi |F_{0}\right)$$

where $E\left(\xi |F_{0}\right)=E\left(\xi \right)/P\left(\Omega_{0}\right)$ (Theorem 1, chapter II §7, [6])

Accordingly, the relation between the two measurable spaces $\left(\Omega_{0},F_{0}\right)$ and (Ω,F) is established:

(5) $$\forall A\in F_{0}\subset F,|Q\left(A\right)=\int_{A}\xi dP/P\left(\Omega_{0}\right)$$

Next, we show how the context can be fitted into the abstract theory such that Eq. (5) holds between the measurable spaces $\left(\Omega^{2},\;F⊗F\right)$ and $\left(\Omega_{0}\times \;\Omega ,F_{0}⊗F\right)$. Essentially, we want to show:

(i) We partition the outcome space finitely.

Suppose (X,Y) is a random vector on $\left(\Omega^{2},\;F⊗F,P\right)$, we partition the outcome space $\Omega^{2}$ based on the images of either entry of (X,Y) (X is chosen here). We partition R into finite intervals. $R=\;\cup_{i=0}^{m}D_{i}$, in which $D_{0}≔\left(-\infty ,d_{0}\right],D_{i}≔\left(d_{i-1},d_{i}\right],D_{m}≔\left(d_{m},\infty \right]$. Set $d_{0}>0$, and then the accidents and near accidents are nested in $D_{0}$. Moreover, the outcome space can be partitioned using the pre-images of $D_{i}$,
  $$\Omega =\cup_{i}\Omega_{i}=:\cup_{i}X^{-1}\left(D_{i}\right)$$

(ii) We can choose a measurable function which satisfies ξ(ω)=1 if $\omega \in F_{0}$ and ξ(ω)=0 if $\omega \notin F_{0}$.

Define a projection $\pi_{0}:\left(\Omega^{2},\;F⊗F\right)\mapsto \left(\Omega_{0}\times \;\Omega ,F_{0}⊗F\right)$ such that:
  $$\pi_{0}\left(\omega \right)=\omega \cdot I\left\{\left(X,Y\right)\left(\omega \right)\in D_{0}\times \;R\right\}$$
Choose $\xi \left(\omega \right)=\pi_{0}\left(\omega \right)$, then $\forall A\in F_{0}⊗F$.
  $$Q\left(A\right)=\int_{A}\pi_{0}dP/P\left(\Omega_{0}\right)=P\left(A\right)/P\left(X^{-1}\left(D_{0}\right)\right)$$

(iii) Q is a probability measure on $\left(\Omega_{0},F_{0}\right)$.

The countable additivity is granted by the fact that P is a probability measure and $\int_{A}\xi dP=P\left(A\right)$ if $A\in F_{0}$, as we define in (ii). The condition that Q(A) integrates to 1 and $Q(\Omega_{0})=1$ is granted since the maximum value $\int_{A}\xi dP$ can take is $P(\Omega_{0}$).

We apply Eq. (5) to establish the relation between the measure P and Q by choosing $A=\left(-\infty ,x\right]\times \left[y,\infty \right)\in F_{0}⊗F$ when $x\leq d_{0}$:

(6) $$P\left(X\leq x,Y>y\right)=Q\left(X\leq x,Y>y\right)\cdot P\left(X^{-1}\left(D_{0}\right)\right)=Q\left(\tilde{X}>\tilde{x},Y>y\right)\cdot P\left(X^{-1}\left(D_{0}\right)\right)$$

By Sklar theorem, there is a survival copula $\bar{C}$ on $\left(\Omega_{0}\times \;\Omega ,F_{0}⊗F,Q\right)$ such that

$Q\left(\tilde{X}>\tilde{x},Y>y\right)=\bar{C}\left(1-F_{1}\left(\tilde{x}\right),1-F_{2}\left(y\right)\right)$

## Adjustment when the sample rank correlation is negative

Some copulas do not support data where the sample has non-positive rank correlations. The problem can be solved by transforming Δv decreasingly. The resulting probability is unchanged owing to the invariance property of copula under monotone transformation. First, we denote the transformed Δv as $\left(\tilde{Y_{1}},\cdots ,\tilde{Y_{n}}\right)≔\left(-Y_{1},\cdots ,-Y_{n}\right)$.

**Proposition**: Let $\left(X_{1},X_{2}\right)$ be a random vector that has a joint distribution $H_{X}\left(x_{1},x_{2}\right)=C_{X}\left(F_{1}\left(x_{1}\right),F_{2}\left(x_{2}\right)\right)$ and marginal distributions $F_{1},F_{2}$. Suppose $T_{1},T_{2}:R\mapsto R$ are monotone decreasing transformations where $Y_{1}=T_{1}\left(X_{1}\right),Y_{2}=T_{2}\left(X_{2}\right),\text{and}\;H_{Y}\left(y_{1},y_{2}\right)=C_{Y}\left(F_{T_{1}}\left(y_{1}\right),F_{T_{2}}\left(y_{2}\right)\right)$, then $C_{Y}\left(u,v\right)=\bar{C}\left(1-u,1-v\right)$.

*proof*: See [7]

Using the above proposition, we can establish a connection between the dependence structure when both margins are transformed and the dependence structure with the original data set.

On the probability space $\left(\Omega_{0}\times \;\Omega ,F_{0}⊗F,Q\right)$, applying the proposition to $F_{X}\left(x\right)-C\left(F_{X}\left(x\right),F_{Y}\left(y\right)\right)$ , we obtain:

$$F_{X}\left(x\right)-C\left(F_{X}\left(x\right),F_{Y}\left(y\right)\right)=F_{X}\left(x\right)-\left(\bar{C}\left(F_{X}\left(x\right),F_{Y}\left(y\right)\right)+F_{X}\left(x\right)+F_{Y}\left(y\right)-1\right)$$

$$=\bar{F_{Y}}\left(y\right)-C^{T}\left(1-F_{X}\left(x\right),1-F_{Y}\left(y\right)\right)$$

$$=F_{T_{2}}\left(T_{2}\left(y\right)\right)-C^{T}\left(F_{T_{1}}\left(T_{1}\left(x\right)\right),F_{T_{2}}\left(T_{2}\left(y\right)\right)\right)$$

Then, the probability of an interaction becomes:

$$P\left(X\leq x,Y>y\right)=\left(F_{X}\left(x\right)-C\left(F_{X}\left(x\right),F_{Y}\left(y\right)\right)\right)\cdot P\left(\Omega_{0}\right)$$

$$\;\;\;\;\;\;\;\;\;\;\;\;\;\;\;\;\;\;\;\;\;\;\;\;\;\;\;\;\;\;\;\;\;=\left(\;F_{T_{2}\left(Y\right)}\left(T_{2}\left(y\right)\right)-C^{T}\left(F_{T_{1}\left(X\right)}\left(T_{1}\left(x\right)\right),F_{T_{2}\left(Y\right)}\left(T_{2}\left(y\right)\right)\right)\;\right)\cdot P\left(\Omega_{0}\right)$$

$$P\left(X\leq x,Y\leq y\right)=C\left(F_{X}\left(x\right),F_{Y}\left(y\right)\right)\cdot P\left(\Omega_{0}\right)$$

$$\;\;\;\;\;\;\;\;\;\;\;\;\;\;\;\;\;\;\;\;\;\;\;\;\;\;\;\;\;\;\;\;\;=\left(C^{T}\left(F_{T_{1}\left(X\right)}\left(T_{1}\left(x\right)\right),F_{T_{2}\left(Y\right)}\left(T_{2}\left(y\right)\right)\right)+1-F_{T_{1}\left(X\right)}\left(T_{1}\left(x\right)\right)\;-\;F_{T_{2}\left(Y\right)}\left(T_{2}\left(y\right))\right)\right)\cdot P\left(\Omega_{0}\right)$$

where $T_{1},T_{2}$ are decreasing transformations for the margins, $F_{T_{1}\left(X\right)}\left(x\right)=1-{\left(1+\gamma \frac{T_{1}\left(x\right)-T_{1}\left(u\right)}{\sigma }\right)}^{-1/\gamma }$, $F_{T_{2}\left(Y\right)}\left(T_{2}\left(y\right)\right)$ can either be an empirical distribution or a parametric distribution, and $C^{T}$is the copula associated with the joint distribution $\left(T_{1}\left(X\right),T_{2}\left(Y\right)\right)$.

## Difference between the presented method and multivariate extreme value theory

The key difference between the multivariate threshold excess model and the proposed copula-based approach lies in the selection of marginal distributions. In the multivariate threshold excess model, all margins must follow an unconditional Generalized Pareto (GP) distribution. However, in the proposed copula approach, this requirement is more flexible—only one or a few margins need to follow the GP distribution. Additionally, the proposed method uses a **conditional** GP distribution, which further distinguishes it by altering the probability space used in the model (details are elaborated in Chen et al. [2]).

## Method validation

The data analysis was performed using R software [8] and several R packages. Namely, “ggplot2” [9] was used to enhance the aesthetics of the plots, “extRemes” [10] was used for univariate extreme value analysis, and “copula” (Jun Yan et al. 2020) was used for parameter estimation of the copulas, computing copula probabilities, and simulating copulas.

The data used in illustrating the proposed method are video footages from roadside camera. Video analysis was performed to extract trajectories and indicators were computed from the trajectories (in total 4772 scenarios of paired trajectories). Along each trajectory, two indicators, the minimum Euclidean distance between two vehicles and the corresponding Δv values were used to describe a traffic interaction. The minimum distance is calculated when two vehicles appeared at the same time in the video, using the formula

$$MD=\min_{i}\sqrt{{\left(x_{1,i}-x_{2,i}\right)}^{2}+{\left(y_{1,i}-y_{2,i}\right)}^{2}}$$

where $\left(x_{1,i},y_{1.i}\right),\left(x_{2,i},y_{2.i}\right)$ are the coordinates of the first and the second vehicle separately at the i th shared frame.

The indicator Δv is calculated through the formula:

$$\Delta v_{1}=\;\frac{m_{2}}{m_{1}+m_{2}}\times \sqrt{v_{1}^{2}+v_{2}^{2}-2v_{1}v_{2}\cos\alpha };\;\;\;\;\;\Delta v_{2}=\;\frac{m_{1}}{m_{1}+m_{2}}\times \sqrt{v_{1}^{2}+v_{2}^{2}-2v_{1}v_{2}\cos\alpha }$$

where *m_1_* and *m_2_* are vehicle masses, *v_1_* and *v_2_* are their speeds, and *α* refers to the approach angle. Herein, the masses of the vehicle are assigned a fixed value depending on whether the vehicle is a passenger car, minivan, or truck. The maximum of the two value is used in analysis.

**Fig. 1:**
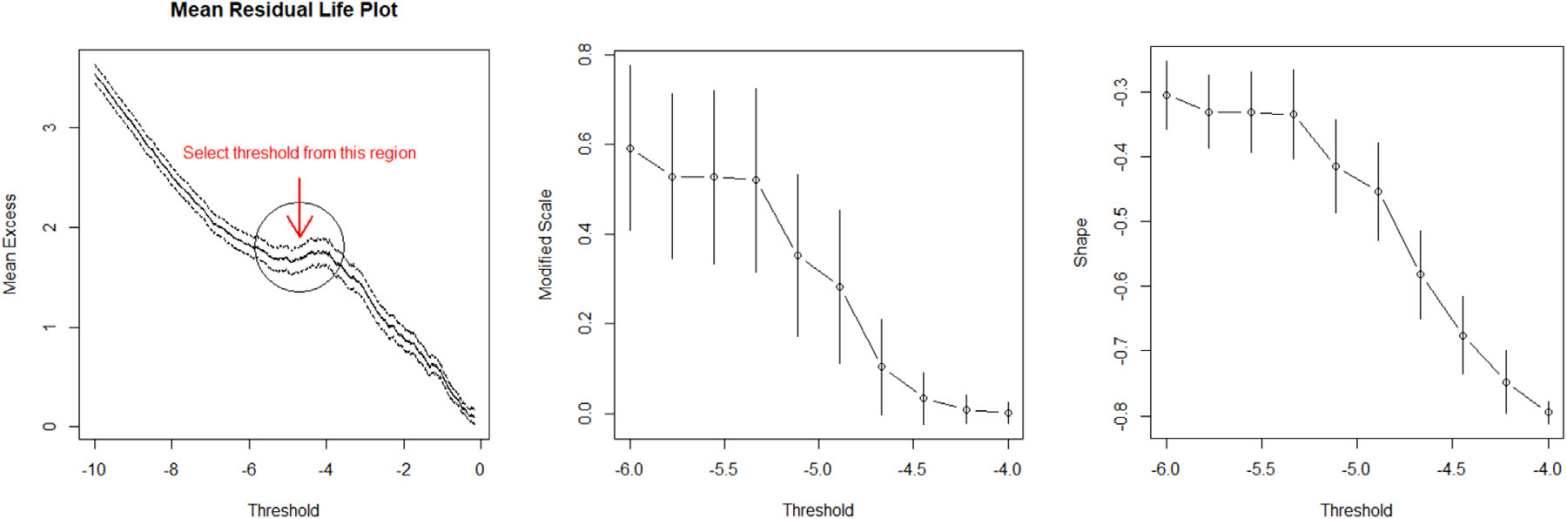
Criteria for threshold selection for Generalized Pareto distribution, the principle is to select threshold that is as large as possible while the mean residual life plot is linear and the stabilities of the parameter estimates are relatively stable.

**Fig. 2:**
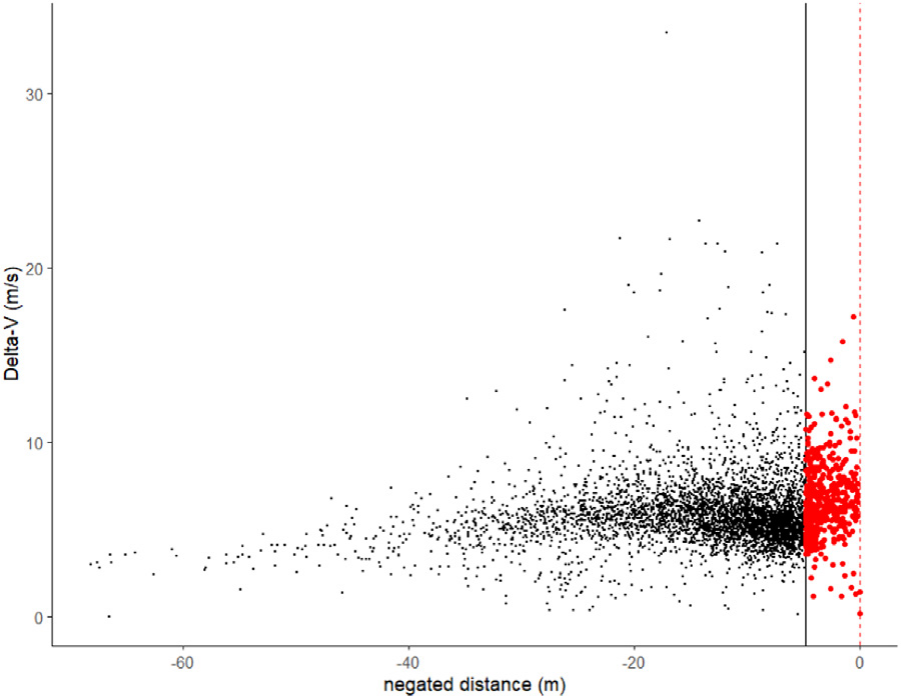
The traffic interactions recorded in terms of minimum distance and Δv. The outcome space $\Omega_{0}$ refers to the right of the black vertical line and the events to be extrapolated is to the right of the red dashed line.

**Fig. 3:**
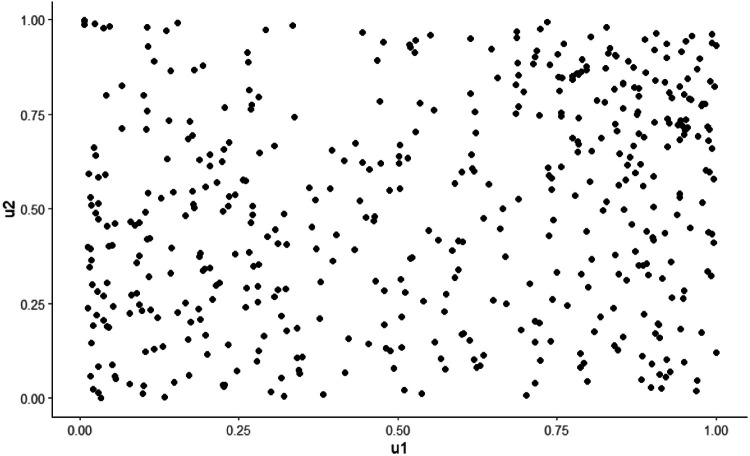
The original data are transformed to uniform observation by $\left(u_{i},v_{i}\right)=\left(F_{1}\left(\tilde{x_{i}}\right),F_{n}\left(y_{i}\right)\right)$.

**Fig. 4:**
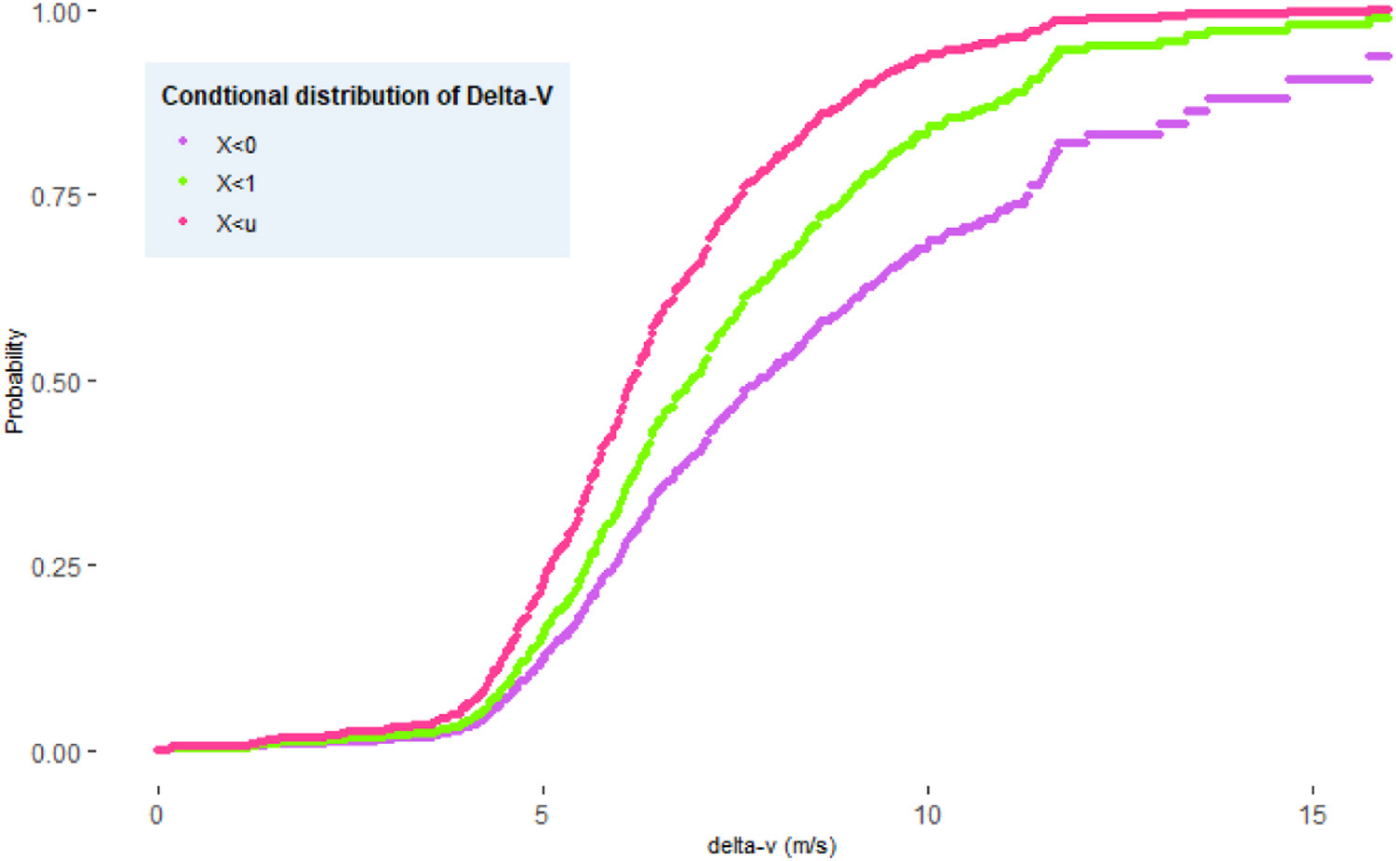
The distribution of Δv when it is conditioned on different proximities.

The near interactions are selected based on the tail behavior of minimum distance (Fig. 1). The traffic interactions can be visualized in the following scatter plot (Fig. 2, Fig. 3, Fig. 4).

We use the inference for margins^3^ and a semi-parametric estimator based on rank correlation to estimate the copula parameters. Prior to the analysis, the proximal indicator must be transformed decreasingly. The simplest decreasing transformation (i.e., negation) was used in this study. The transformed distance is denoted as $\left(\tilde{X_{1}},\cdots ,\tilde{X_{n}}\right)≔\left(T_{1}\left(X_{1}\right),\cdots ,T_{1}\left(X_{n}\right)\right)$. The implementation is outlined in five steps.

1. *Apply the peaks over threshold (POT) model to the margin of proximity*: apply univariate POT to determine the upper-tail distribution of $\tilde{X}$. The upper-tail marginal distribution of transformed distance is approximated using Eq. (4). The result is:
  $$F\left(\tilde{x}\right)=1-{\left(1-0.493\cdot \frac{\tilde{x}+4.84}{2.60}\right)}^{1/0.493}$$
2. *Transform the marginal distribution into a standard uniform distribution*: first, we restrict the outcome space to $\Omega_{0}$ by excluding observations whose distances do not exceed the chosen threshold. The corresponding Δv values are kept unchanged. The uniform observations $\left(u_{1},v_{1}\right),\cdots ,\left(u_{m},v_{m}\right)$ are obtained using $\left(u_{i},v_{i}\right)=\left(F_{1}\left(\tilde{x_{i}}\right),F_{2}\left(y_{i}\right)\right)$, in which m is the number of observations that exceed the threshold, and $F_{2}$ is the distribution function for the interaction severity indicator on $\Omega_{0}$. We use the empirical distribution $F_{n}\left(y\right)=\frac{1}{m}\sum_{i=1}^{m}I\left\{Y_{i}\leq y\right\}$.
3. *Fit the copula to the transformed data*: the uniform observations $\left(u_{1},v_{1}\right),\cdots ,\left(u_{m},v_{m}\right)$ are used to fit different copula functions. The rank approximate estimator based on Kendall τ is used here. For one-parameter copulas with parameter θ, the rank correlation τ is defined as
  $$\tau =4\cdot E\left(C_{\theta }\left(U,V\right)\right)-1=4\int \int_{{\left[0,1\right]}^{2}}C_{\theta }\left(u,v\right)dC_{\theta }\left(u,v\right)-1=:g\left(\theta \right)$$
  g:R↦[−1,1]. Moreover, if the function g is bijective such that $g^{-1}\left(\tau \right)$ is continuously differentiable with respect to τ, then the estimator $\hat{\theta_{n}}=g^{-1}\left(\hat{\tau }\right)$ is a weakly consistent estimator of θ, where $\hat{\tau }$ is the sample estimate of τ (Theorem 1, [11]).
  $$\hat{\tau }=\frac{2n}{n-1}\sum_{i=2}^{n}\sum_{j=2}^{n}\left(C_{n}\left(u_{i},v_{j}\right)\cdot C_{n}\left(u_{i-1},v_{j-1}\right)-C_{n}\left(u_{i},v_{j-1}\right)\cdot C_{n}\left(u_{i-1},v_{j}\right)\right)$$
  where $C_{n}\left(u,v\right)=n^{-1}\sum_{i=1}^{n}I\left\{U_{\left(i\right)}\leq u,V_{\left(i\right)}\leq v\right\}$ is the empirical copula, and $U_{\left(i\right)},V_{\left(i\right)}$ are equal to the rank of the i^th^ observation divided by n.
4. *Selection of the models*: compare the non-parametric and parametric estimates from different models using the goodness of fit. We selected three single-parameter copula which captures the upper tail dependence (Gumbel copula), symmetric dependence (Normal copula), and lower tail dependence (Clayton copula). The expression are presented in Table 1.

**Table 1:** Parameter estimates for different copula models. The domain of the parameter is written in parenthesis.

Copula	Model form	Estimated parameter (Domain)	Test statistics	p-value
Gumbel	$C\left(u,v\right)=exp\left(-{\left({\left(-\ln(u)\right)}^{\theta }+{\left(-\ln(v)\right)}^{\theta }\right)}^{1/\theta }\right)$	θ=1.169 (θ∈[1,∞))	0.033	0.248
Normal	$C\left(u,v\right)=F\left(\sigma_{1}\cdot \Phi^{-1}\left(u\right),\sigma_{2}\cdot \Phi^{-1}\left(v\right)\right)$*	ρ=0.225 (ρ∈[−1,1])	0.047	0.001
Clayton	$C\left(u,v\right)=\max{\left(u^{-\theta }+v^{-\theta }-1,0\right)}^{-1/\theta }$	θ=0.338 (θ∈(0,∞))	0.06	0.002

⁎ Φ is the c.d.f. of standard Gaussian, F is a bivariate normal distribution with a covariance matrix whose diagonal entries are $\sigma_{1},\sigma_{2}$ and off-diagonal entries are ρ.
  Greater magnitude of the parameter value indicates strong dependence. Note that Gumbel and Clayton copula support only data with positive dependence.
5. *Compute the probability*: the probability of an interaction is evaluated by plugging in different values of x,y into Eq.6. The conditional distribution of Δv is computed combing Eq.1, Eq.4, and Eq.6. Since only Gumbel copula has passed the goodness of fit test, the computation is based on Gumbel copula.

## Limitations

The limitation of our method is primarily related to its stability:

1. **Stability of the Generalized Pareto margin**: When the shape parameter of the Pareto distribution falls below −0.5, the maximum likelihood estimator faces convergence issues. In such cases, extrapolating from the Generalized Pareto distribution becomes unreliable, and the resulting extreme events should not be used as a basis for conditioning.
2. **Copula parameter estimation**: Estimating the copula parameters sometimes requires carefully fine-tuned starting values, which can be difficult to determine. While non-parametric method, like the one we employed, provide an alternative solution, they are limited to single-parameter copulas. A further issue with non-parametric estimation is that it does not produce a maximized log-likelihood value, which means model selection based on information criteria such as AIC (Akaike Information Criterion) is impossible.

## Ethics statements

No animal or human subject was involved in this study, and the subject was identifiable from the video data.

## CRediT authorship contribution statement

**Zhankun Chen:** Conceptualization, Formal analysis, Writing – original draft. **Carl Johnsson:** Software, Data curation, Visualization. **Carmelo D'Agostino:** Conceptualization, Funding acquisition, Writing – review & editing.

## Declaration of competing interest

The authors declare that they have no known competing financial interests or personal relationships that could have appeared to influence the work reported in this paper.

## Data Availability

Data will be made available on request.

## Footnotes

1 An accident is defined when the spatial proximity between two motor vehicles becomes zero.

2 Sklar's result applies also to survival function, such that there exists$\;\bar{C}\left(1-F_{1}\left(x\right),1-F_{2}\left(y\right)\right)=P\left(X>x,Y>y\right)$ whereas $C\left(u,v\right)=\bar{C}\left(1-u,1-v\right)+u+v-1\langle /END\rangle$It means that the parameter of the margin and copula parameter are estimated separately.

3 It means that the parameter of the margin and copula parameter are estimated separately.

## References

[1] Zou Y., Zhang Y. (2016). A copula-based approach to accommodate the dependence among microscopic traffic variables. Transportation Research Part C: Emerging Technologies.

[2] Chen Z., Yastremska-Kravchenko O., Laureshyn A., Johnsson C., D'agostino C (2024). Stochastic method based on copulas for predicting severe road traffic interactions. Anal. Methods Accid. Res..

[3] Sklar M. (1959). Fonctions de répartition à n dimensions et leurs marges. Annales de l'ISUP.

[4] Pickands J. (1975). Statistical inference using extreme order statistics. The Annals of Statistics.

[5] Širjaev A. (2016).

[6] Širjaev A. (2016).

[7] Nelsen R. (2006).

[8] R Development Core Team 2023.R: a language and environment for statistical computing. https://www.R-project.org/.

[9] Wickham H. (2016).

[10] Eric G., Richard W. (2016). ExtRemes 2.0: an extreme value analysis package in R. J. Stat. Softw..

[11] Tsukahara H. (2005). Semiparametric estimation in copula models. Canadian Journal of Statistics.

